# Mesenchymal stem cells and exosomes improve cognitive function in the aging brain by promoting neurogenesis

**DOI:** 10.3389/fnagi.2022.1010562

**Published:** 2022-10-18

**Authors:** Xiaowen Zhang, Xuejia Hou, Liu Te, Zhou Zhongsheng, Jinlan Jiang, Xiaodong Wu

**Affiliations:** Scientific Research Center, China-Japan Union Hospital of Jilin University, Changchun, China

**Keywords:** mesenchymal stem cell, exosome, brain aging, cognitive function, inflammation, neurogenesis

## Abstract

Biologically speaking, normal aging is a spontaneous and inevitable process of organisms over time. It is a complex natural phenomenon that manifests itself in the form of degenerative changes in structures and the decline of functions, with diminished adaptability and resistance. Brain aging is one of the most critical biological processes that affect the physiological balance between health and disease. Age-related brain dysfunction is a severe health problem that contributes to the current aging society, and so far, there is no good way to slow down aging. Mesenchymal stem cells (MSCs) have inflammation-inhibiting and proliferation-promoting functions. At the same time, their secreted exosomes inherit the regulatory and therapeutic procedures of MSCs with small diameters, allowing high-dose injections and improved therapeutic efficiency. This manuscript describes how MSCs and their derived exosomes promote brain neurogenesis and thereby delay aging by improving brain inflammation.

## Introduction

Aging is a topic that has fascinated scientists and philosophers throughout history (da Costa et al., 2016). As the global population is aging, the prevention of brain aging is a common problem. Aging is associated with a progressive decrease in the effectiveness of mechanisms that maintain homeostasis of the body and its organs and tissues, which leads to an increased risk of various pathologies and death (Isaev et al., 2019). At present, China has entered an aging society, and it is expected that in 2050, 30% of the total population will be over 60 years old, The problem of aging is becoming increasingly severe. It is common in science to think of human aging as a set of characteristics that change over time and to refer to someone as “older” or “younger” (Ferrucci et al., 2018). Brain aging is a significant cause of most neurodegenerative diseases and is often irreversible and lacks an effective treatment, leading to a dramatic decline in quality of life (Poulose et al., 2017). As with other organ systems, brain function gradually declines during the aging, mainly in learning and memory functions (Mattson and Arumugam, 2018). Cognitive dysfunction can be described as an imbalance in the structural and functional organization of the brain at all three levels: the molecular/cellular level, the local circuit level, and the large-scale network level. Each of these levels interacts dynamically with the others and exhibits the characteristics of an open complex system (Birle et al., 2021). Cognitive function is complex and may also be affected by diet, and adequate nutrition is effective in preventing cognitive decline (Martínez García et al., 2018). Therefore, since the development of medicine, scientists have been working to explain the phenomenon of cognitive decline in the elderly.

Some studies point out that age-related cognitive decline is characterized by a considerable reduction or even death of neurons in the brain (Foster, 2006; Wang et al., 2021). In the hippocampus (and perhaps in other brain areas), neuronal death can partially compensated by neuronal generation. However, neuronal production is significantly impaired with age (Isaev et al., 2019). In the adult mammalian hippocampus, new neurons are derived from the stem and progenitor cell divisions, a process known as adult neurogenesis (Cope and Gould, 2019). Neurogenesis occurs throughout life in the ventricular-subventricular zone (V-SVZ) of the lateral ventricles and the subgranular zone (SGZ) of the hippocampal dentate gyrus (DG) (Apple et al., 2017). Neurogenesis plays a critical role in neuroplasticity, brain homeostasis, and central nervous system (CNS) maintenance. It is essential to maintaining cognitive function and repairing damaged brain cells affected by aging and brain disease (Poulose et al., 2017). Adult hippocampal neurogenesis directly impacts cognitive function since hippocampal formation is closely linked to the storage and processing of memory (Bekinschtein et al., 2010; Araki et al., 2021). In recent years, evidence has accumulated that neurogenesis can restore a more youthful state during aging. In addition, increased adult neurogenesis contributes to a variety of human diseases, including cognitive impairment and neurodegenerative diseases (Niklison-Chirou et al., 2020). The appearance of neurodegenerative diseases (including Alzheimer’s and Parkinson’s) increases exponentially with age (Grimm and Eckert, 2017), so aging is considered to be the most critical risk factor for almost all neurodegenerative diseases (Saha and Sen, 2019).

**FIGURE 1:**
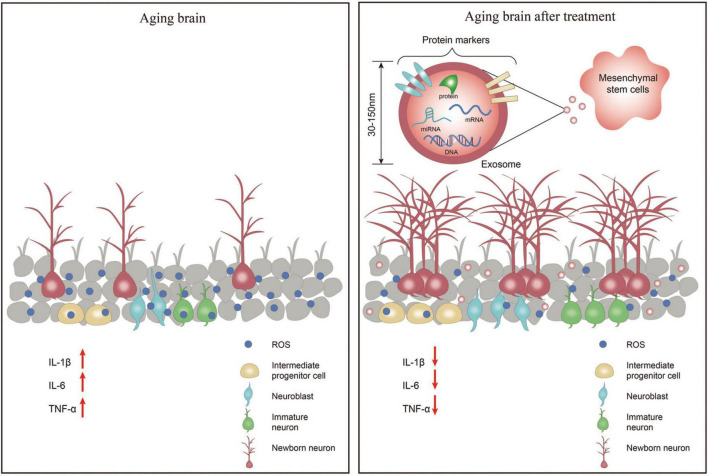
In the aging brain, the number of neuronal cells is significantly reduced, the levels of inflammatory factors IL-1β, IL-6, and TNF-α are increased leading to neuroinflammation, and the levels of reactive oxygen species (ROS) are increased causing oxidative stress in the brain. After treatment with exosomes secreted by mesenchymal stem cell-extracellular vesicle (MSC-EV), the number of neuronal cells increased, the levels of inflammatory factors IL-1β, IL-6, and TNF-α decreased, and the levels of ROS decreased thereby reducing oxidative stress in the brain.

Neurogenic inflammation is triggered by neural activation, resulting in neuropeptide release, rapid plasma extravasation and edema, leading to conditions such as headaches. Neuroinflammation is a local inflammation of the peripheral nervous system (PNS) and CNS (Matsuda et al., 2019). Neuroinflammation has been shown to alter neurogenesis in adults. Various inflammatory components, such as immune cells, cytokines, or chemokines, regulate neural stem cells’ survival, proliferation, and maturation (Sung et al., 2020). During normal brain aging, increased inflammatory activity is caused by the activation of glial cells (Jin et al., 2021). It has been shown that mesenchymal stem cells (MSCs) can stimulate neurogenesis and angiogenesis and delay neuronal cell death (Dabrowska et al., 2019). At the same time, their secreted exosomes are smaller in size and cause less immune response in the body, which is a hot topic of current research (Figure 1).

## Mechanisms and manifestations of brain aging

Cellular senescence is an important factor in tissue deterioration and the accumulation of senescent cells is considered a hallmark of and a pathological cause of aging (Tominaga and Suzuki, 2019). Among the organelles most closely related to senescence is the nucleus (Niedernhofer et al., 2018), mitochondria (Campisi et al., 2019), and lysosomes (Levine and Kroemer, 2019; Wong et al., 2020). The core is mainly involved in the cell cycle, telomere, and epigenomic changes (Pal and Tyler, 2016). A new study finds that age-related epigenetic changes can be reversed by interventions (Kane and Sinclair, 2019); Mitochondria are mainly involved in oxidative stress due to the increase of reactive oxygen species (ROS) and mutations in mitochondrial DNA (mtDNA), inflammation, and apoptosis (Jang et al., 2018), which are important factors that induce the onset of aging; In lysosomes, it was found that lysosomes and lysosome-related organelles play an important role in the regulation of aging and longevity (Soukas et al., 2019), which is mainly associated with autophagy (Wong et al., 2020); in the cytoplasmic matrix and extracellular, etc., are primarily involved in signaling pathways related to inflammation and fibrosis (Jiang et al., 2017), such as Meschiari et al. (2017) who stated that cardiac fibrosis is usually one of the hallmarks of cardiac aging. These signaling pathways release inflammatory factors and chemokines that contribute to the deterioration of the senescent cells’ microenvironment, which transmits aging signals and affects the transformation of surrounding healthy cells into senescent cells.

The brain is the most complex and vital human organ (Derbyshire, 2018; Hussain et al., 2019; Benito-Kwiecinski and Lancaster, 2020), consuming more energy than any other tissue in proportion to its size. Microstructural degeneration of the gray and white matter in the human brain during aging leads to tissue softening and tissue atrophy (Blinkouskaya et al., 2021). The rate of brain atrophy during aging can predict whether someone will develop cognitive impairment and dementia, and analysis of cross-sectional histological sections suggests that atrophy is the combined result of dendritic regression and neuronal death (Mattson and Arumugam, 2018). Some scholars have used magnetic resonance imaging (MRI) to find that the frontal, parietal, and temporal lobes decrease with age (Deelchand et al., 2020) while the frontal, parietal, and temporal lobes control language, memory, auditory (Sur and Golob, 2020), motor (Singhal et al., 2020), and attention functions of the human brain. Initially, these aging mechanisms occur mainly at the cellular level due to slowed metabolic activity and ischemia, such as inflammation, mitochondrial dysfunction (Stefanatos and Sanz, 2018), oxidative stress (Lushchak, 2021), and calcium dysregulation (Chandran et al., 2019), but then gradually manifest themselves in tissue and eventually organ-level changes in brain shape (Blinkouskaya et al., 2021). In addition, some environmental factors can affect the rate of structural changes in the brain during aging. For example, adequate aerobic exercise increases hippocampal volume, effectively improving memory function (Erickson et al., 2011); overweight and obesity can lead to hippocampal atrophy and affect brain health (Cherbuin et al., 2015).

## Mesenchymal stem cell and exosome properties

Mesenchymal stem cells, officially named 29 years ago, represent a class of cells in the human and mammalian bone marrow (BM) and periosteum that can be isolated and expanded in culture while maintaining their ability to be induced to form a variety of different cells *in vitro* (Caplan, 2017). MSCs have a solid proliferative capacity (Naji et al., 2019) and can self-renew and differentiate into tissue-specific cells (e.g., osteoblasts, chondrocytes, and adipocytes), and therefore have great potential in regenerative medicine (Samsonraj et al., 2017; Fu et al., 2019). In addition to its pluripotency, MSC has immunomodulatory properties and has been investigated as a potential treatment for various immune diseases (Li and Hua, 2017). MSCs influence most immune effector cells through direct contact with immune cells and local micro-environmental factors. According to studies, the immunomodulatory effects of MSC are mainly delivered through cytokines secreted by MSC. However, apoptotic and metabolically inactivated MSCs have recently shown immunomodulatory potential, with regulatory T cells and monocytes playing a pivotal role (Song et al., 2020). Secondly, since MSCs do not express significant histocompatibility complexes and immunostimulatory molecules, they are not detected by immune surveillance and do not cause graft rejection after transplantation, which is a significant breakthrough point for regenerative medicine (Han et al., 2019). Various animal models (myocardial infarction mice, burned mice, and diabetic mice) and clinical trials have shown that MSCs show good results in repairing damaged tissues (Oh et al., 2018; Selvasandran et al., 2018; Xu et al., 2018). MSCs have the homing ability, which means they can migrate to the site of injury and secrete some growth factors, cytokines, and chemokines that are beneficial for tissue repair (Zhang S. J. et al., 2016; Ullah et al., 2019). Many experimental studies in ischemic stroke have shown that MSCs are able to modulate immune responses and play a neuroprotective role by stimulating neurogenesis, oligodendrogenesis, astrogliogenesis, and angiogenesis. MSCs may also have the ability to replace damaged cells, but paracrine factors released directly into the environment or *via* extracellular vesicles (EVs) appear to play the most significant role (Dabrowska et al., 2019).

Exosomes are nanoscale vesicles (30–150 nm in diameter) secreted by most cells (Xunian and Kalluri, 2020). They are surrounded by a lipid bilayer and carry a variety of biomolecules, including proteins, lipids, metabolites, RNA, and DNA. When exosomes are taken up by other cells, these exosomes are transferred and affect the phenotype of the recipient cells. Exosomes play a crucial role in bioactive molecule transport, immune response, antigen presentation, protein regulation, cellular homeostasis, and extracellular matrix remodeling (Mori et al., 2019). Thus, exosomes are considered to be an essential mediator of intercellular communication (Wortzel et al., 2019). MSC-derived exosomes contain cytokines, growth factors, lipids, and messenger RNA (mRNA) and regulate microRNAs (miRNAs) function (Phinney and Pittenger, 2017). Exosomes mainly act on the organism in a paracrine manner (Mori et al., 2019), and it has been established that the mode of action of the therapeutic effect of stem cells is mainly paracrine mediated by stem cell secretory factors (Ha et al., 2020), so it is presumed that exosomes primarily act during stem cell therapy. Exosomes have a relative therapeutic effect on a variety of diseases. In a mouse model of acute kidney injury (AKI), MSC-derived exosomes (MSC-Exo) accumulated mainly in inflamed kidneys, whereas in a brain hemorrhage model, MSC-Exo was detected in the injured brain (Harrell et al., 2019). Two proteins commonly found in exosomes, CD81 and tumor susceptibility gene 101 (TSG101), have been confirmed by Western blot (Riazifar et al., 2019; Vakhshiteh et al., 2019). Exosome therapy has shown similar therapeutic effects to direct MSC transplantation without causing multiple adverse outcomes. The complexity of the integrated function of its contents improves the therapeutic effect of MSC-Exo (Zhang B. et al., 2016). Multiple studies have found that after intravenous administration of exosomes, they are predominantly distributed in vascular-rich organs and organs associated with the reticuloendothelial system, such as the liver, lungs, spleen and kidneys (Aimaletdinov and Gomzikova, 2022). In addition, Xu et al. (2020) found that DIR (lipophilic, near-infrared fluorescent anthocyanine dye)-labeled exosomes could be detected in mouse brain after intravenous injection of exosomes by near-infrared fluorescence (NIRF).

## Therapeutic effects of mesenchymal stem cells and exosomes on brain aging

### Mesenchymal stem cells repair neuronal cells in the hippocampus to slow brain aging

Aging is a natural process; the most obvious outward manifestation accompanying brain aging is a decline in cognitive function. The structure to consider for cognitive decline in the brain is necessarily the hippocampus-a brain region known to play an essential role in learning and memory consolidation as well as in affective behavior and emotion regulation and whose functional and structural plasticity [e.g., neurogenesis (Boldrini et al., 2018)] occurs in adulthood. Neurobiological changes seen in the aging hippocampus, including increased oxidative stress and neuroinflammation, altered intracellular signaling and gene expression, and reduced neurogenesis and synaptic plasticity, are thought to be associated with age-related declines in cognitive function (Bettio et al., 2017). In animal experiments, the Morris water maze is usually used to test the learning memory ability of mice. In contrast, after intracerebroventricular injection of MSC of human BM origin, the aged MSC-treated group showed significant improvements in spatial memory accuracy and prolonged persistence in single- and three-hole target areas as demonstrated in the Morris water maze compared with the aged control group. MSC treatment increased the number of neuroblasts in the hippocampal DG, decreased the number of reactive microglia, and restored presynaptic protein levels compared to older controls. And after MSC transplantation, MSCs mainly migrated to the DG, CA1, and CA3 regions of the hippocampus. Cognitive deficits are associated with altered levels of several neurological factors, such as brain-derived neurotrophic factor (BDNF), nerve growth factor (NGF), and glial cell-derived neurotrophic factor (GDNF) (Budni et al., 2015). In contrast, human MSCs express a variety of neuromodulators that promote neuronal survival and neurogenesis (Crigler et al., 2006). We can see this result in the hematoxylin–eosin (HE) pathology and Nissl staining of the MSC-treated and senescent control groups. This experiment concluded that intracerebroventricular injection of human bone marrow-derived mesenchymal stem cells (hBM-MSCs) was effective in improving spatial memory in aged rats and that the treatment improved some of the functional and morphological brain characteristics that are typically altered in aging rats (Zappa Villar et al., 2019).

### Mesenchymal stem cells slow brain aging by promoting angiogenesis

Gervois et al. (2016) discussed that the neuroprotective effect was mainly attributed to soluble factors secreted by stem cells. Furthermore, it has been shown that stem cells can form vascular structures and secrete pro-angiogenic factors *in vitro*, positively influencing the growth of blood vessels *in vitro* and *in vivo* (Gervois et al., 2016). For example, cerebral ischemia is the most common disease in the elderly (Lappin et al., 2017). Age is the main unmodifiable risk factor for cerebral ischemia. When located in the inflammatory microenvironment *in vivo*, MSC can release a variety of angiogenic and neurotrophic factors as well as anti-inflammatory molecules; in addition, MSC appears to have an excellent homing ability when administered by systemic routes. Several studies have shown that bone marrow-derived stem cell transplantation in the peripheral circulation improves neurological function and reduces infarct volume. Cellular therapies using MSCs can enhance endogenous repair mechanisms in the damaged brain by supporting the processes of neoangiogenesis, neurogenesis, and neural reorganization. The mechanism by which MSCs improve infarcted brain tissue appears to be more related to the ability of MSCs to release neuroprotective factors (a paracrine mechanism) than to their ability to replace (Sandu et al., 2017).

### Mesenchymal stem cells and its secreted exosomes slow brain aging by suppressing the expression of pro-inflammatory factors

Aging is characterized by developing a persistent pro-inflammatory response (Franceschi et al., 2007), and the aging brain is also susceptible to inflammation (Jauhari et al., 2020). Yet, inhibiting of pro-inflammatory factor expression alleviates cognitive impairment in the brain (Yan et al., 2022). Microglia are resident immune cells of the CNS and play a key role in maintaining brain homeostasis (Aguzzi et al., 2013). In the aging brain and neurodegeneration, microglia lose their homeostatic molecular signature and can promote increased production of pro-inflammatory cytokines (Marschallinger et al., 2020). Studies have demonstrated that reactive microglia infiltrate the hippocampus in aging rats and cause it to exhibit an inflammatory state (Pardo et al., 2017). MSC can maintain the resting phenotype of microglia or control microglia activation by producing multiple factors (Yan et al., 2013), thereby controlling the inflammatory response and delaying brain aging. Studies have shown that exosomes obtained from MSC secretion also have anti-inflammatory effects. It regulates the brain infiltration of leukocytes and thus protects the nerves (Wang et al., 2022). Inflammatory responses have long been associated with neurodegenerative processes. And TNF-α, IL-1β, and IL-6, cytokines that support inflammation, are significantly increased in the aging brain. Cui et al. (2019) found that the pro-inflammatory regulators TNF-α, IL-1β, and IL-6 were significantly reduced in the brains of aging mice after MSC-Exo treatment. In the Morris water maze test, the MSC-Exo-treated group significantly reduced escape latency from 3 days after acquisition training, and learning and memory abilities were significantly improved in mice treated with MSC-Exo. Thus, MSC-derived exosomes can rescue memory deficits by modulating the inflammatory response (Cui et al., 2019). Data suggest that MSC-derived exosomes can enter microglia and inhibit their activation to shift them back to function, thereby suppressing inflammation and promoting recovery of brain function (Chen et al., 2020).

### Mesenchymal stem cells and its secreted exosomes slow brain aging by promoting neurogenesis

Recent studies have shown the neurogenesis in the adult brain (de Godoy et al., 2018). In addition, the treatment of MSCs has been shown to stimulate neurogenesis in the rat brain and is proposed to be implemented in a number of neurodegenerative diseases (Gobshtis et al., 2017; Kho et al., 2018). Some scientists have also used exosomes secreted by MSCs for treatment and found that exosomes promote neurogenesis in the subventricular zone (SVZ) and DG of the hippocampus and reduce cognitive impairment associated with Parkinson’s disease, stroke, and traumatic brain injury (Yang et al., 2017; Zhang et al., 2017). From the preceding, it is known that MSC-derived exosomes disrupt the polarization of M1 microglia and trigger their transition to the M2 phenotype, thereby significantly reducing inflammation. In addition, exosome treatment is neuroprotective against oxidative stress and also expands neuronal nerve density (Reza-Zaldivar et al., 2019). Several studies provide evidence that exosomes interact with neurogenic ecotopes through miRNA transfer to neural precursor cells, triggering neural remodeling events, neurogenesis, angiogenesis, and synaptogenesis (Cheng et al., 2018). Some scientists have also modified exosomes with miRNAs. For example, Chen et al. (2021) found that elevated miR-26a enhanced axonal growth in hippocampal neurons and axonal regeneration in the PNS, and then they overexpressed miR-26a in exosomes and found that it could activate the mammalian target of rapamycin (mTOR) pathway to enhance axonal growth and renewal in the nervous system, thus promoting neurogenesis (Table 1).

**TABLE 1:** Application of mesenchymal stem cell (MSC) and exosome in brain aging.

Type	Mechanism of action	References
hBM-MSCs	Improve brain aging by repairing nerve cells in the hippocampus	(Budni et al., 2015)
MSCs	Improve brain aging by promoting angiogenesis	(Gervois et al., 2016)
MSCs	Maintain the resting phenotype of microglia or 220 control microglia activation by producing multiple factors	(Marschallinger et al., 2020)
MSC-Exo	Regulates the brain infiltration of leukocytes and thus protects the nerves	(Pardo et al., 2017)
MSC-Exo	Inhibit microglia activation to shift them back to function	(Wang et al., 2022)
MSC-Exo	Neuroprotective against oxidative stress and also expands neuronal nerve density	(Zhang et al., 2017)
MSC-Exo	Overexpressed miR-26a in exosomes could activate the mTOR pathway to enhance axonal growth and renewal in the nervous system, thus promoting neurogenesis	(Reza-Zaldivar et al., 2019)

## Discussion

Based on the data collected, it is known that brain aging subsequently increases the incidence of neurodegenerative diseases, which seriously affect the quality of life of the elderly. Scientists have been seeking better ways to slow down aging, and MSCs and their derived exosome therapies are emerging promising strategies for treating various diseases. In recent years, there has been increasing research interest in exosomes, their innate ability to transport genetic material, protect it from cellular degeneration, and deliver it to recipient cells in a highly selective manner, suggesting that MSC-derived exosomes are an ideal delivery system for small molecules and a means of gene therapy for cancer treatment and potentially regenerative drugs. In addition, encouraging preclinical data suggest that MSC-derived exosome therapies may be superior to cell-based therapies in terms of safety and versatility. Today, the preparation of MSCs has become proficient and the extraction of exosomes is being refined. However, the technology for purification of exosomes after extraction still needs to be addressed. Furthermore, the progression of exosomes on tumors has been widely reported in the last decade or so. For example, MSC-derived exosomes from bone marrow (BM MSCs) stimulate the hedgehog signaling pathway in osteosarcoma and gastric cancer cell lines, thereby promoting tumor growth (Vakhshiteh et al., 2019). Therefore, the translation of therapies from the laboratory to the clinic requires a clear understanding of component characterization, immune response, etc., in order to optimize their clinical application.

## Author contributions

JJ and XW contributed to the conception and design. XZ wrote the manuscript and figure. XH collected the data and designed the figure. LT and ZZ performed literature search and provided valuable comments. All authors contributed to the article and approved the final manuscript.
